# The risk factors for the recurrent upper gastrointestinal hemorrhage among acute peptic ulcer disease patients in Syria: A prospective cohort study

**DOI:** 10.1016/j.amsu.2022.103252

**Published:** 2022-01-15

**Authors:** Sara Mona Bitar, Maen Moussa

**Affiliations:** Department of Gastroenterology, Faculty of Medicine, University of Aleppo, Syria

**Keywords:** Acute peptic ulcer, Upper gastrointestinal bleeding, Rebleeding, Risk factors, Blood transfusion

## Abstract

**Background:**

Upper gastrointestinal bleeding (UGIB) is a life-threatening medical emergency characterized by bleeding from the esophagus, stomach, or duodenum. This study aims to analyze the risk factors for upper gastrointestinal tract rebleeding among acute peptic ulcer patients.

**Methods:**

This is a cohort clinical study conducted between July 2018 and June 2020. Patients admitted or hospitalized because of UGIB or developed it during their hospital stay were included.s The patients were divided into two groups for the statistical analysis using Forrest's ulcer rebleeding risk classification. Group 1: Forrest 1a+1b+2a+2b, and group 2: Forrest 2c+3. The fasting time before the endoscopic procedure was from 12 to 24 hours. Follow-ups were conducted for 30 days after the treatment.

**Results:**

The total number of included subjects was 152, out of which 57.89% (n = 88) were male patients. The mean SD for patients’ age was 52.63 16.89±; more than 40% (n = 62) of subjects were using antiplatelet medications, while only 13.15% (n = 20) used NSAIDs, and the mean SD for the transferred units was 2.32 ± 1.88, 7.24% (n = 11) of patients died. After 30 days of the treatment, 6.57% (n = 10) of patients suffered from recurrent bleeding. The most common presentation was melena 67.95% (n = 103), 53% (n = 81) of patients had hematemesis, 69.73% (n = 106) patients had gastric ulcer and 30.26% (n = 46) had duodenal ulcers.

**Conclusion:**

Age, NSAIDs, altered mental capacity, Forrest classification (Ia,Ib, and IIa), and blood transfusion were associated with a higher risk of rebleeding. Furthermore, patients who needed 3.83 blood units were at higher risk of recurrent bleeding.

## Highlights

• Characteristics of upper gastrointestinal rebleeding in Syria.
• Most important risk factors that were associated with high rebleeding risk.
• The amount of blood transfusion needed among rebleeding upper gastrointestinal patients.

## 1 Introduction

Upper gastrointestinal bleeding (UGIB) is a common medical emergency that requires immediate attention and hospitalization. It is also responsible for around 2–10% death annual deaths worldwide [1]. It is usually referring to bleeding from the esophagus, stomach, or duodenum. Common signs and symptoms of upper GI bleeding include hematemesis, melena, haematochezia, hemodynamic instability, lower haemoglobin values. Some of the clinical presentations might be delayed; therefore, any patients with melena often will need a blood transfusion [2]. Furthermore, research has shown that the mortality rate among patients with melena is lower than patients with hematemesis [3].

The sudden upper gastrointestinal bleeding after successful treatment is considered recurrent bleeding or treatment failure [4]. The highest risk for recurrent bleeding among peptic ulcer patients after successful endoscopic treatment is usually in the first 72 hours. Those patients should be put on a high dose of proton pump inhibitor (PPI) drugs at least for the first 72 hours. In the past, The rate of recurrent gastric bleeding among peptic ulcer patients was around 30%. However, the rapid advancement in endoscopic treatment procedures has reduced the rate of recurrent bleeding to around 10% [5].

The recurrent upper gastrointestinal hemorrhage has many signs and symptoms. The specific signs of recurrent bleeding may include: hematemesis or passing the blood through the nasopharyngeal passages within 6 hours of the endoscopic procedure, haematochezia after a brief period of normal stool passing, recurrent melena, sudden drop in blood pressure (below 90 mm Hg systolic, high pulse rate (above 110)), at least two degrees drop in blood hemoglobin after two normal readings, and a persistent decrease in blood hemoglobin associated with haematochezia or melena [6,7]. General signs may include; dizziness or faintness, exhaustion and weakness, looking pale, chest tightness, anuria or oliguria, and cramps [5,8,9].

In the past few decades, many improvements have been made in the management of upper gastrointestinal bleeding. However, many patients have high risk of treatment failure or rebleeding. This study aims to detect and analyze the risk factors for upper gastrointestinal tract rebleeding among acute peptic ulcer patients after the bleeding has been stopped. The risk factor assessment can also help stratify patients based on the risk of rebleeding to high and low risk, enabling health care providers to predict who needs urgent endoscopy and who can be safely discharged with a low risk of rebleeding.

## 2 Methods

### 2.1 The study setting and patients

This is an observational clinical cohort study conducted between July 2018 and June 2020 at the gastroenterology unit of the Internal Medicine Department, Aleppo University Hospital, Syria. Patients who were admitted or hospitalized because of upper gastrointestinal bleeding or developed upper GI bleeding during their hospital stay were included. The patients were divided into two groups using Forrest*'s* ulcer rebleeding risk classification. Group 1 (37 patients): Forrest 1a+1b+2a+2b, and group 2 (115 patients): Forrest 2c+3, and the total number of included subjects was 152. This study followed the STROCSS criteria and its guidelines [10,11]. This study was ethically approved and registered at the University of Aleppo research registry with the following number: 26-07-2018/5077 and was registered in the Research Registry with the following ID “researchregistry7395”[12]. All patients were properly informed about the study details before they joined the study, and informed consent was obtained from all of them. The ethical committee of the Faculty of Medicine at the University of Aleppo has reviewed and approved this manuscript before publication.

### 2.2 Data collection

The required information about each patient was recorded using a data collection form. The form was divided into multiple sections. Section one/Personal and demographic information: age, gender, marital status, address, average income, and habits. Section two/Medical history: Risk factors, Medication history, laboratory test results, vitals and biomarkers. Section three/Patients’ clinical presentation: Presented signs and symptoms that were associated with the upper gastrointestinal bleeding (like melena, hematemesis, epigastric pain etc.), the onset of bleeding, results of the endoscopy, laboratory results (blood hemoglobin/hematocrit, CBC, blood electrolyte, X-ray results, blood group and urinary test results, ultrasound test results to investigate the liver and Portal vein etc).

### 2.3 The endoscopic procedure

All endoscopic procedures were performed in the gastroenterology division of the internal medicine department at Aleppo university hospital. Peptic ulcer was determined by the presence of more than 5 mm break in the inner lining of the mucosal membrane of the stomach and duodenum, and then all cases were stratified according to Forrest ulcer classification [13]. The fasting time before the endoscopic procedure was from 12 to 24 hours. Follow-ups were conducted for 30 days after the treatment.

### 2.4 Inclusion and exclusion criteria

Inclusion criteria:

• The presence of upper gastrointestinal bleeding due to peptic ulcer
• Patients of both gender (male and female)
• Age group from 18 to 70
• All Admitted and referred patients for GI endoscopy during the study period

Exclusion criteria:

• Did not agree to participate in the study
• Missing data, dropout and lost to follow-up
• The presence of other factors that can lead to gastric bleeding other than peptic ulcer
• Patients with cancerous diseases.
• Canceled endoscopy procedures for any reason
• COVID-19 Patients

### 2.5 Sample size

The sample size was calculated using Fleiss's formula for cohort studies for an expected odds ratio between 10% and 30% based on the available literature and a 95% confidence interval [14]. After continuity correction, the total sample size for the given odds ratios was 68 and 22, respectively. In this study, we were able to recruit 152 subjects.

### 2.6 Statistical analysis

Microsoft Excel (2019) was used to arrange and organize the collected data. The statistical analysis was conducted using the Statistical Package for the Social Sciences (SPSS) (Version 22.0, 2015; IBM Inc., Chicago, IL, USA). Descriptive statistics were done to identify the nature of the study population. The independent sample *t*-test was used to compare the means in continuous variables such as age, while categorical variables were tested using Chi-square. Also, One-way analysis of variance (ANOVA) was used to determine the statistically significant difference between the means of three or more continuous independent variables, and the ‘post hoc Tukey test’ is used to identify outliers. The adopted statistical significance cut-off point was at *p < 0.05*.

## 3 Results

The total number of included subjects was 152, out of which 57.89% (n = 88) were male patients. The sociodemographic characteristics of the recruited sample are described in Table 1. The mean SD for patients’ age was 52.6316.89±; more than 40% (n = 62) of subjects were using antiplatelet medications, while only 13.15% (n = 20) used NSAIDs. Patients who needed blood transfusion represented 67.11% (n = 102) of the total sample size, and the mean SD for the transferred units was 2.32 ± 1.88, 7.24% (n = 11) of patients died. After 30 days of the treatment, 6.57% (n = 10) of patients suffered from recurrent bleeding.

**Table 1:** The medical and sociodemographic characteristics of the recruited sample.

	Number/Value	Standard Deviation/Percentage
**Male**	88	57.89%
**Female**	64	42.11%
**Age (yr)**	52.63	16.89±
**Smoking**	79	51.97%
**Antiplatelet**	62	40.78%
**NSAIDs**	20	13.15%
**Systolic blood pressure (mm Hg)**	90.28	29.69±
**Diastolic blood pressure (mm Hg)**	68.36	30.87±
**Pulse rate (beats/min)**	85	30±
**Hemoglobin (gm/dL)**	9.11	1.95±
**Platelets count (platelets per microliter)**	245.56	125.72±
**Blood Urea (mg/dL)**	46.83	11.34±
**Serum Albumin (g/dL)**	3.35	1.21±
**GCS Score (below 15)**	11	7.24%
**Blood transfusion**	102	67.11%
**Number of transferred blood units**	2.32	1.88±
**Recurrent Bleeding (30 days)**	10	6.57%
**Number of deaths**	11	7.24%

**Fig. 1:**
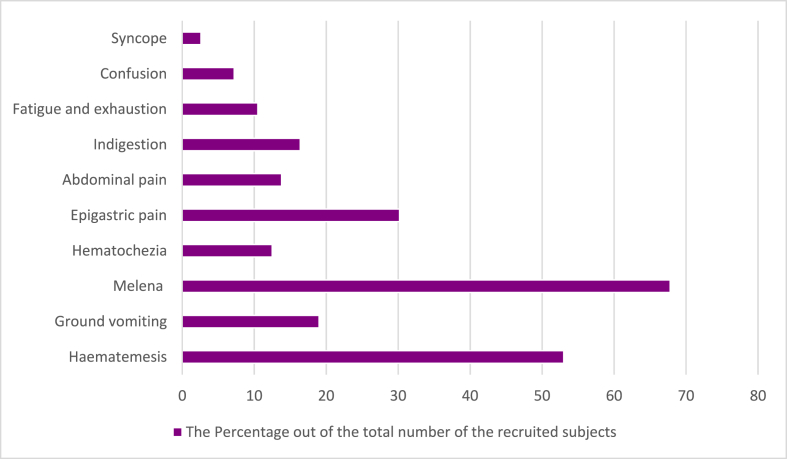
The distribution of clinical GI bleeding signs and symptoms.

Patient's were presenting to the hospital with multiple clinical manifestations for upper GI bleeding, Fig. 1. The most common one was melena, appearing in 67.95% (n = 103) of cases, 53% (n = 81) of patients had hematemesis, epigastric pain was the third most common sign with 46 (30.34%) cases, and 29 (19.23%) patients had ground vomiting. In terms of mental capacity, 11 (7.23%) patients were confused, and only 4 (2.63%) suffered from syncope.

Esophagogastroduodenoscopy's findings have shown that almost 70% of patients had gastric ulcers. In addition, gastritis appeared in 14.47% of patients, fresh blood was detected in the stomach of 7.89% of participants, while the other findings did not exceed 2% of the recruited sample. On the other hand, 42.71% of patients were suffering from duodenal ulcers, and fresh blood in the duodenum was found only in 1.97% of the cases.

Based on the endoscopic observations, most patients had gastric ulcer 69.73% (n = 106) and 30.26% (n = 46) had duodenal ulcers, Table 2. According to Forrest classification, almost two-thirds of patients were classified as clean-based ulcers patients [III: 64.47%(n = 98)], and almost 10% were actively bleeding lesions [Ia: 5.26% (n = 8), and 4.61%(n = 7)], Table 2.

**Table 2:** The observed characteristic of the ulcers after endoscopy based on Forrest classification.

	Number	Percentage
**The ulcer's site**		
Gastric	106	69.73%
Duodenal	46	30.26%
**Forrest classification**		
Ia	8	5.26%
Ib	7	4.61%
IIa	10	6.58%
IIb	12	7.89%
IIc	17	11.18%
III	98	64.47%

Multiple risk factors for recurrent bleeding were statistically significant, Table 3. Age, the use of non-steroidal anti-inflammatory drugs, patients who had any score below 15 on the Glasgow coma scale, lesions based on Forrest classification (Ia,Ib, and IIa), and blood transfusion. The number of transferred blood units was significantly higher among the patients with recurrent bleeding (*p* = 0.003). Furthermore, patients who needed more than 3.83 blood units showed a significant association with recurrent bleeding (*p* = 0.009).

**Table 3:** The risk factors for recurrent bleeding among upper gastrointestinal bleeding patients after 30 days of the treatment.

Variable	Recurrent Bleeding Patients (N = 10)		Non-Recurrent bleeding Patients (N = 142)		*p*-Value
	Value/Number	Standard Deviation/Percentage	Value/Number	Standard Deviation/Percentage	
**Gender (Male)**	6	60%	82	57.75%	0.761
**Age (yr)**	51.74	±12.24	59.76	±10.45	0.003*
**Smoking**	7	70%	72	50.70%	0.166
**Anti-platelets**	6	60%	56	39.44%	0.195
**NSAID**	5	50%	15	10.56%	0.005*
**Systolic BP**	88.15	±23.35	93.65	±22.43	0.287
**Pulse Rate**	84	±28	87	±31	0.429
**Hemoglobin rate**	9.87	±2.13	8.89	±1.4	0.336
**INR**	1.13	±0.86	1.20	±0.87	0.539
**Serum Albumin**	3.2	±1.43	3.3	±1.71	0.574
**Blood Urea Nitrogen**	45.81	±12.47	44.63	±14.19	0.638
**Creatinine**	1.24	±0.89	1.38	±0.66	0.316
**GCS Score (Below 15)**	2	20%	9	6.36%	0.012*
**Forrest (Ia,Ib,IIa)**	5	50%	20	14.08%	0.006*
**Blood transfusion**	10	100%	92	64.79%	0.003*
**Number of transferred units**	3.83	±1.42	2.29	±2.17	0.009*

**Fig. 2:**
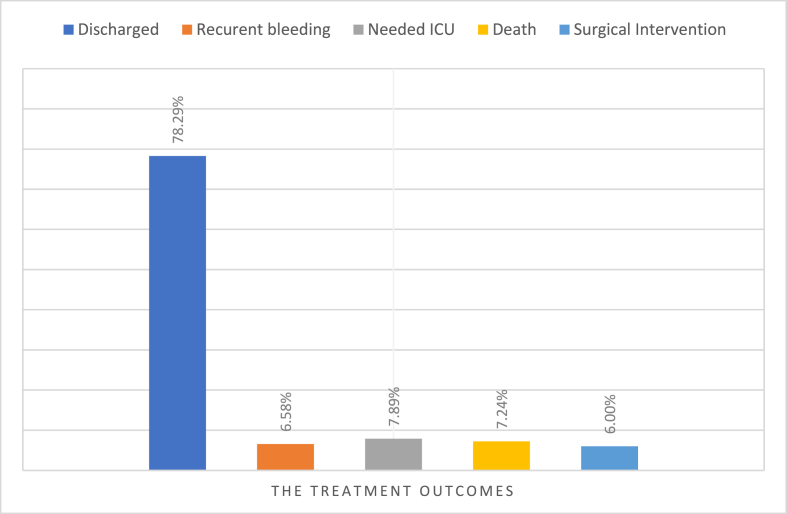
Treatment outcomes for the included subjects during the study period.

The vast majority of patients were discharged with no complications during the study's follow-up period, Fig. 2. However, 6.58% (n = 10) patients suffered from recurrent bleeding with in less than 30 days, 7.89% (n = 12) needed ICU admission, and 7.24% (n = 11) died. The total number of patients who needed surgical intervention during the study represented 6% (n = 9) of all cases.

## 4 Discussion

This study described the risk factors for recurrent bleeding in 30 days among 152 upper GI ulcers patients. Surprisingly, patients who suffered from recurrent bleeding were significantly younger than those who did not, which can be explained by the high rates of NSAIDs and Aspirin users among patients with recurrent bleeding. A recent review has shown that NSAIDs could cause severe damage in the upper and lower GIT; they found that upper GI lesions were present in up to 50% of patients taking NSAIDs, and bowel injuries affect 70% of the chronic users [15]. Furthermore, they found that the risk of peptic ulcers among patients on NSAIDs was four times higher than the non-user group.

In our study, an impaired or reduced consciousness on GCS was associated with a higher risk of recurrent bleeding. Similar to our work, the univariate analyses in Chandnani et al.’s work has shown that the Glasgow coma scale below 14 significantly increased the risk for bleeding (*p* = 0.001). However, unlike our study, serum albumin was significantly lower among UGIB patients with recurrent bleeding (*p* = 0.008) [16]. Furthermore, the most common presentation was hematemesis showing up in almost all patients, and the mean SD for patients age was 43.5 ± 17.2, which is significantly lower than our study. In a large Chinese multicentre study (1416 patients) assessing the UGIB, GCS scores were significantly lower than our study (score ≤10). This study has included neurocritical care patients, which explains the low GSC scores. Unlike our study, the use of anticoagulants was significantly associated with recurrent bleeding [17,18].

More severe cases in the Forrest classification were associated with upper gastrointestinal rebleeding. Similar to our findings, the highest rebleeding rate was in Forrest Ia peptic ulcers (59%). De Groot and his group have found that the Forrest classification showed more reliable predictive power for peptic ulcers than duodenal ulcers [19]. However, it failed in mortality prediction. A group of researchers from Korea has found that Forrest classification was significantly associated with recurrent bleeding, while Rockall score was significantly associated with mortality. The odds were 39 times higher of rebleeding in Forrest Ia-b and 29 times Forrest IIa, while mortality risks were more than nine times in cases with Rockall score 4–6 and 101 times in cases with scores above 7 [20]. In another study, the rebleeding cases accounted for 9% of the subjects included and 14.8% for those who had a successful endoscopic treatment. Additionally, recurrent bleeding was more common in duodenal ulcer patients than gastric ulcers (11.9% VS 4.0%, p = 0.004), and Forrest classification was associated with rebleeding was significantly related to a higher mortality rate [21].

Our study has shown that the number of needed blood units to be transfused was associated with recurrent bleeding. In a multicentre international study that included patients from three continents, the required blood units to be transferred was averaged around 3, and that is lower than our finding [22], which can be attributed to the longer time required to control bleeding in our study due to the limited available resources. Interestingly, they found that the previous use of Antithrombotic Agents was associated with reduced mortality rates and reduced hospital stay in patients with High-risk Upper Gastrointestinal Bleeding [22,23].

Some studies have suggested some kidneys disease's indicators such as blood urea nitrogen creatinine ratio as predictors for recurrent bleeding. Wu et al. have found that a BUN/Cr ratio of >30 was an independent risk factor for UGIBs and a useful indicator for pre-endoscopic assessment [24]. In a ten-year retrospective cohort study, Kumar and her co-researchers suggested that the high rates of blood urea in the last 24 hours can be used for predicting GI bleeding since acute GI bleeding can be associated with acute kidney failure [25]. However, BUN's results showed that high BUN was not significantly associated with recurrent bleeding during our study. A stable hemodynamic is an essential key factor in maintaining a healthy kidney function among patients with upper GI bleeding [26,27], and disturbed hemostasis was associated with an increased c-reactive protein rate and a higher risk of rebleeding in UGIB patients [28]. The recurrent bleeding cases in our study represented 6.57%, which is a low rate compared to other studies. In a Canadian study, 14.1% of acute upper gastrointestinal hemorrhage patients suffered from recurrent bleeding [29]. While the death rate was close to our study, 7.24%, ranging from 7.4% to 11% [30,31]. However, in another study, the death rate was significantly lower with 0.7% only [32]. Recurrent bleeding was associated with high death rates and a significant increment in hospital admissions [33].

This study presents a precious insight into the characteristics and the nature of risk factors for rebleeding among upper gastrointestinal peptic ulcer patients. It is one of few studies from a warzone, and it fills up some of the scientific gaps since there is a lack of studies from Syria. Furthermore, the sample size was adequate and gave a proper presentation for the nature of the presented condition. However, one major limitation of this study was the limited available treatment options due to the ongoing conflict in Syria. The only available treatment option was 10 mL diluted epinephrine injections (1:10000) and PPIs IV infusions. And this is due to the war circumstance, lack of supply, not to mention the overstretched health care system, and burnt health care workers (HCW) due to the COVID 19 outbreak. In fact, HCW burnout during the pandemic is fairly common in both developed and less developed countries [34,35].

## 5 Conclusion

Among hospitalized UGIB patients, many risk factors were associated with recurrent bleeding during 30 days of follow-ups. Most importantly, Forrest's classification (Ia, Ib, and IIa) and the need for blood transfusion (3.8 units) were associated with increased recurrent bleeding among acute upper gastrointestinal ulcer patients in Syria. More research is required to identify other risk factors.

## Ethical approval and consent to participants

This study was ethically approved and registered at the University of Aleppo research registry with the following number: 26-07-2018/5077. All patients were properly informed about the study details, and proper informed consent was obtained before they joined the study. The ethical committee of the Faculty of Medicine at the University of Aleppo has reviewed and approved the publication of this research project.

## Consent for publication

All patients who agreed to participate in this study were informed about the publication. A copy of the written consent form is available for review by the editor of this journal.

## Funding acknowledgement

This study received no funding or sponsorship from any entity.

## Ethical approval

This study was ethically approved by the ethical committee of the faculty of medicine and registered at the University of Aleppo research registry with the following number: 26-07-2018/5077. The ethical committee of the Faculty of Medicine at the University of Aleppo has also reviewed and approved the publication of this research project.

## Author contribution

SMB: concept, data collection, analysis, and draft write-up. MM: Interpretation, supervision, and critical revision of the article.

## Registration of research studies

1. Name of the registry: Research Registry
2. Unique Identifying number or registration ID: researchregistry7395
3. Hyperlink to your specific registration (must be publicly accessible and will be checked): https://www.researchregistry.com/browse-the-registry#home/registrationdetails/619e066eb359be001f488f6c/

## Guarantor

Dr. Maen Moussa and Dr. Sara Mona Bitar.

## Provenance and peer review

Not commissioned, externally peer-reviewed.

## Declaration of competing interest

No Any.

## Data Availability

All the data that was generated or analyzed in the study are included in the published article.
